# The multi-level influence mechanism of family background on children’s education level: The moderating effect of socioeconomic status

**DOI:** 10.1371/journal.pone.0323477

**Published:** 2025-05-14

**Authors:** Ting Qin, Pingqiang Wei, Yuanyuan Xie

**Affiliations:** School of Literature and Journalism, Xihua University, Chengdu, Sichuan Province, China.; National center for chronic and non-communicable diesease prevention and control, CHINA

## Abstract

**Background:**

A superior family background often provides children with more educational resources and a better learning environment, while poor or educationally deficient families may put children at a disadvantage on the starting line of education. This difference is not only related to the fate of individuals, but also to the fairness and justice of the whole society. Therefore, this study explores the influence mechanism of family background on children’s education level and the moderating effect of socioeconomic status.

**Methods:**

Based on the data of China General Social Survey in 2021, a total of 4592 samples were selected. Principal component analysis model, hierarchical regression model and hierarchical linear model were used to verify the relationship between family background, socioeconomic status and children’s education level.

**Results:**

(1) Family background has a significant positive impact on children’s education level (*β*=1.266; *p* < 0.001). (2) Socioeconomic status positively regulates the influence of family background on children’s education level (*β*=0.274; *p* < 0.001). Specifically, even if the parents’ education level is low, families with high socioeconomic status can make up for their disadvantages through economic investment, or obtain scarce educational resources through social relations. On the contrary, even if parents are highly educated, they are limited by economic constraints or lack of social capital, and it is difficult to translate family cultural advantages into actual educational investment (such as inability to bear the cost of extracurricular training or inability to obtain admission to prestigious schools), which limits the improvement of children ‘s education level. (3) Regional and urban-rural differences significantly affect the allocation of educational resources.

**Conclusion:**

Family background affects children’s education level and is regulated by socioeconomic status. At the same time, regional and urban-rural differences affect the distribution of educational resources. We call for attention to the construction of family background and encourage families to create a good educational environment for their children. Increase investment in education for families and regions with lower socioeconomic status, and promote the balanced distribution of educational resources in different regions and between urban and rural areas to promote educational equity.

## 1 Introduction

In the course of the development of human society, children, as the basic unit of the family, not only carry the continuation of blood, but also place their parents’ expectations for the future and the hope of society. Education equips children with knowledge, skills, and the tools to compete effectively in their future endeavors. Children with good education often have stronger ability to adapt and solve problems, and can be more flexible in the complex and changeable social environment. At the same time, education is also a key way to cultivate children’s correct values, outlook on life and world outlook, which helps them form a sound personality and noble moral sentiment [1–3]. When we look at the current situation of children’s education level, it is not difficult to find the challenges and inequalities. Multiple factors such as family, region, economy and culture are intertwined, which jointly affect the opportunity and quality of children’s education. In some areas where resources are scarce or ideas are backward, children’s educational rights and interests are often difficult to be fully guaranteed, which is undoubtedly a major obstacle to the overall progress of society. Previous studies have focused on the important role of family background in children’s education level, but the research is not comprehensive and in-depth. There are few studies on whether socioeconomic status has a moderating effect. After the Chinese government promulgated and implemented the “double reduction” policy [4], these issues are particularly important. Therefore, based on the data of China Comprehensive Social Survey in 2021, this study uses principal component analysis model, hierarchical regression model and hierarchical linear model to explore the impact of family background on children’s education level in Chinese family education, and reveals the moderating effect.

Family background factors include parents’ education level, occupation, income, culture and social status. These factors will affect the family’s emphasis on children’s education, support and educational resources. Among them, the education level of parents is considered to be one of the most important factors. Studies have shown that the higher the level of education of parents, the higher the level of education of children [5–6]. This is because well-educated parents are better able to provide quality educational resources and nurture to their children, while also giving them better educational guidance and support [7–9]. Family background has an impact on children’s enrollment probability [10–11], academic performance [12], and choice of subjects and majors [13–14]. In addition, parents’ emphasis on education affects their children’s educational choices and achievements [15–16]. Families with higher social status can often use their social resources to provide their children with a broader space for education and development. Based on this, this study proposes hypothesis 1: Family background positively affects children’s education level.

As an important family factor, family socioeconomic status affects the education level of children. Families with higher socioeconomic status tend to be able to provide better educational resources [17–18], such as quality schools, rich extracurricular activities, etc. These resources play a key role in children’s development and learning [16,19]. On the contrary, families with lower socioeconomic status may not be able to provide sufficient educational support for their children due to financial constraints, resulting in children being at a disadvantage in educational competition. In addition, family socioeconomic status can affect children’s academic performance [20,21], learning engagement [22], improving personal subjective well-being [23–25], stimulating fertility desire [26], and increasing employment opportunities [27,28]. Based on this, this study proposes hypothesis 2: Socioeconomic status has a moderating effect on the relationship between family background and children’s education level. Specifically, families with higher socioeconomic status, even if faced with unfavorable family background factors, may provide better educational opportunities for their children through economic support and social resources, thus alleviating the negative impact of unfavorable factors on their children’s education level. Conversely, families with lower socioeconomic status, even with favorable family background factors, may not be able to fully support their children’s education due to limited resources.

In China, the allocation of educational resources is a complex and multidimensional problem, which involves many aspects, such as region, urban and rural areas, school type and so on. From the regional level, there is a significant imbalance in the distribution of educational resources in China. Due to the developed economy and large government investment, the eastern region is relatively rich in educational resources, perfect in school facilities and strong in teachers. In the central and western regions, especially in remote rural areas, educational resources are relatively scarce, school conditions are poor, and teachers are weak [29]. This regional difference leads to the inequality of educational opportunities and affects the overall quality of education. The difference between urban and rural areas is also a major problem in the allocation of educational resources in China [30,31]. Due to the advantages of economy, culture and other aspects in urban areas, educational resources are relatively concentrated, school facilities are advanced, and the quality of education is high. In rural areas, due to economic backwardness, insufficient government investment and other reasons, educational resources are scarce, school conditions are poor, and the quality of education is difficult to guarantee [32,33]. This difference between urban and rural areas further aggravates the unfairness of education. Based on this, this study proposes hypothesis 3: Regional and urban-rural differences affect the allocation of educational resources. This hypothesis holds that the differences in economy, culture, policy and other aspects between regions and urban and rural areas are important reasons for the uneven distribution of educational resources. The distribution of educational resources affects the aggregation of groups with different family backgrounds and socioeconomic status, and ultimately affects the level of education of children.

## 2 Data sources and research methods

### 2.1 Data sources

**Fig 1:**
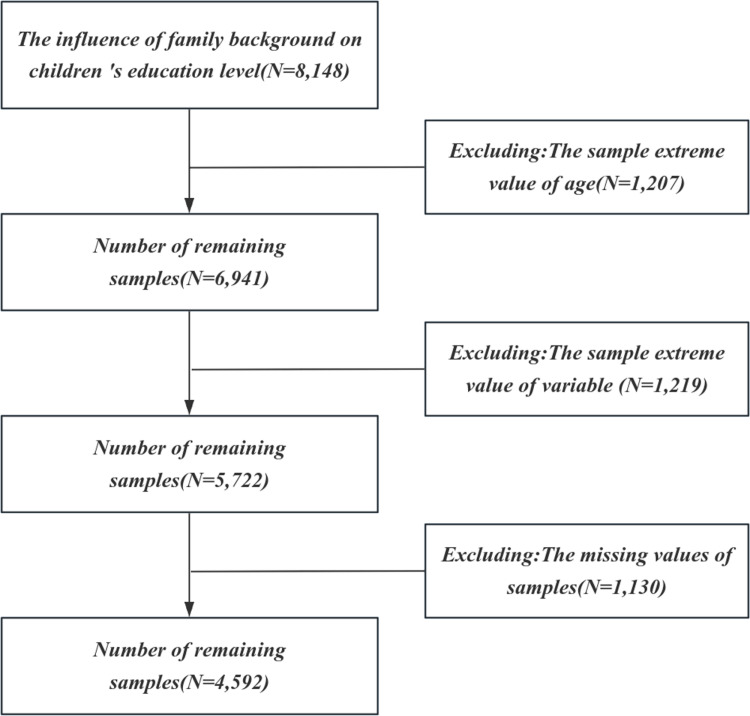
Data screening and preprocessing flow chart.

In order to further explore the influencing factors of the education level of Chinese offspring, this study selected the data of the 2021 China General Social Survey (hereinafter referred to as CGSS) for analysis. CGSS began in 2003, is China ‘s first national, comprehensive, continuous large-scale social survey project. The studies involving humans were approved by the Ethics Committee of Renmin University of China. The studies were conducted in accordance with the local legislation and institutional requirements. Oral consent was obtained due to logistical constraints during the COVID-19 pandemic. Participant consent was recorded digitally, ensuring adherence to ethical guidelines approved by the Ethics Committee of Renmin University of China. The initial sample size of CGSS data in 2021 is 8148, including 700 variables. According to the research needs, we carefully screened and preprocessed these original data, and finally obtained 4592 valid research sample data. Data screening and preprocessing steps are shown in Fig 1.

### 2.2 Description of variable measurement

Parental education levels were measured using two CGSS (2021) questions: A89b (father’s highest education) and A90b (mother’s highest education). Responses ranged from 1 (‘no education’) to 13 (‘postgraduate and above’), with higher scores reflecting greater attainment.

Parental work background were measured using two CGSS (2021) questions: A89g (what is the type of work unit or company your father works?) and A90g (what is the type of unit or company your mother works?). Responses ranged from 1 (‘party and government organs’) to 6 (‘no unit/ self-employment (self-employed)’). The lower scores, the stronger the impact of parents’ work units on children’s education level.

Family economic status were measured using two CGSS (2021) questions: A89g (what is the type of work unit or company your father works?) and A64 (what is the economic status of your family in your locality?). Responses ranged from 1 (‘far below average level’) to 5 (‘far above average level’), with higher scores reflecting greater attainment.

Socioeconomic status was measured using one CGSS (2021) question: A43e (what is your socioeconomic status?). Responses ranged from 1 (‘lower layer’) to 5 (‘upper layer’), with higher scores reflecting greater attainment.

Children’s education level was measured using one CGSS (2021) questions: A7a (yours highest education). Responses ranged from 1 (‘no education’) to 13 (‘postgraduate and above’), with higher scores reflecting greater attainment.

### 2.3 Model technique

#### 2.3.1 Principal component analysis (PCA) model method.

Principal component analysis (PCA) was employed to reduce data dimensionality, assuming linear relationships and sufficient inter-correlation among variables. This ensured the extraction of primary components reflecting family background dimensions.

In this study, there are three original variables: parents’ education level $X_{1}$, parents’ working background $X_{2}$ and family economic status $X_{3}$. First, we need to standardize these three variables to eliminate the influence of dimension and order of magnitude. The standardized Equation is as follows:


$$Z_{j}=\frac{X_{j}-\mu_{j}}{\sigma_{j}},\;j=1,2,3$$ (1)

In Equation (1), $\mu_{j}$ is the mean value of variable $X_{j}$, and $\sigma_{j}$ is its standard deviation. The mean value of the standardized variable $Z_{j}$ is 0, and the standard deviation is 1.

Secondly, the covariance matrix Σ of the standardized variables is calculated. The covariance matrix is a square matrix, whose elements represent the covariance between the various standardized variables. The calculation Equation is as follows:


$$\Sigma =[\begin{array}{ccc} Cov(Z_{1},Z_{1}) & Cov(Z_{1},Z_{2}) & Cov(Z_{1},Z_{3})\\ Cov(Z_{2},Z_{1}) & Cov(Z_{2},Z_{2}) & Cov(Z_{2},Z_{3})\\ Cov(Z_{3},Z_{1}) & Cov(Z_{3},Z_{2}) & Cov(Z_{3},Z_{3}) \end{array}]$$ (2)

In Equation (2), the diagonal element of the covariance matrix is the variance of each variable. Because it has been standardized, the diagonal element should be close to 1, and the non-diagonal element is the covariance between variables.

Then, the eigenvalues and eigenvectors of the covariance matrix Σ are solved. The eigenvalues ($\lambda_{1},\lambda_{2},\lambda_{3}$) and the corresponding eigenvectors ($V_{1},V_{2},V_{3}$) satisfy the following Equation:


$$\Sigma V_{i}=\lambda_{i}V_{i},i=1,2,3$$ (3)

In Equation (3), $\lambda_{1}\geq \lambda_{2}\wedge \lambda_{2}\geq \lambda_{3}\wedge \lambda_{3}\geq 0$.

According to the size of the eigenvalues, the corresponding eigenvectors are used as the first principal component, the second principal component, and the third principal component. The first principal component $F_{1}$ is a linear combination of standardized variables, and its Equation is as follows:


$$F_{1}=V_{1}^{\prime }Z=v_{11}Z_{1}+v_{12}Z_{2}+v_{13}Z_{3}$$ (4)

Here $V_{1}^{\prime }=(v_{11},v_{12},v_{13})$ is the transpose of the eigenvectors corresponding to the first principal component.

Finally, in this study, we extract the three variables, and the extracted three principal components, a new variable will be used as a comprehensive family background index. The principal component score of this new variable combines the information of parents’ education level, parents’ work background and family economic status, and retains the variation in the original variable to the greatest extent, thus simplifying the model and improving the accuracy of the analysis. This new variable will be used in subsequent hierarchical regression analysis to explore the influence mechanism of family background on children’s education level.

#### 2.3.2 Hierarchical regression (moderating effect) model method.

In this study, we will use the hierarchical regression model to explore the impact of family background (a comprehensive variable obtained by principal component analysis, representing the combined effects of parents’ education level, parents’ work background and family economic status) on children’s education level, and pay special attention to the moderating effect of socioeconomic status. The hierarchical regression model allows us to gradually introduce independent variables in order to better understand the unique contribution of each variable to the dependent variable, and to examine how the moderating variable changes the relationship between the independent variable and the dependent variable.

It should be noted that the variable of family background explored in this study is a variable that combines the three dimensions of parents’ education level, professional background and family economic status through principal component analysis (PCA). Among them, family economic status only reflects the current economic capacity of the family (such as income and consumption level), which belongs to the static resource index. The subsequent socioeconomic status variable is used as a moderating variable. Its connotation goes beyond the family’s economic status and covers the family’s position in the class structure (such as professional prestige, social network, policy resource acquisition ability), which belongs to the dynamic resource transformation ability index. Therefore, there are obvious theoretical boundaries and measurement dimensions between family background variables and socioeconomic status variables.

First of all, this study constructs a basic regression model to analyze the direct impact of family background on children’s education level. The mathematical Equation is as follows:


$$EduLevel=\beta_{0}+\beta_{1}FB+\in$$ (5)

In the Equation (5), EduLevel represents the education level of children as the dependent variable of this study; FB is a comprehensive variable of family background obtained by principal component analysis (PCA) as an independent variable; $\beta_{0}$ is the intercept term, $\beta_{1}$ is the regression coefficient of family background, and ∈ is the error term.

Secondly, in order to test the moderating effect of socioeconomic status, this study introduces socioeconomic status SES as a moderating variable in the model. The mathematical Equation of the adjustment effect is extended as follows:


$$EduLevel=\beta_{0}+\beta_{1}FB+\beta_{2}SES+\beta_{3}(FB\times SES)+\in$$ (6)

In Equation (6), SES represents socioeconomic status as a moderating variable; FB×SES is the interaction between family background and socioeconomic status, which is used to capture the moderating effect. $\beta_{2}$ and $\beta_{3}$ are the regression coefficients of socioeconomic status and interaction, respectively.

The existence of moderating effect means that the influence of family background on children’s education level may change with the change of socioeconomic status. If the regression coefficient $\beta_{3}$ of the interaction term is significantly non-zero, then we can say that socioeconomic status plays a moderating role between family background and children’s education level. Through this hierarchical regression model method, we can understand the influence mechanism of family background on children’s education level more comprehensively, and reveal the important moderating effect of socioeconomic status.

#### 2.3.3 Hierarchical linear model method.

The Hierarchical Linear Model (HLM) is a statistical technique used to process data with a nested structure in which low-level units (e.g., individuals) are nested within high-level units (e.g., regions). In this study, HLM was used to explore whether regional and urban-rural differences affect the allocation of educational resources under different socioeconomic status levels.

In this study, there are two layers of data: the first layer is the individual layer, and the second layer is the group layer (region and urban and rural). At the individual level, this study focuses on the educational resources of individual samples, which are mainly affected by socioeconomic status. At the group level, this study focuses on the allocation of educational resources in regions and urban and rural areas.

First of all, this study defines the following three variables:

$Y_{ij}$: the distribution of educational resources (dependent variable) in the ith unit of the jth region (or urban and rural).

$X_{ij}$: the socioeconomic status of the i unit in the jth region (or urban and rural areas) (the first level of independent variables).

$Z_{ij}$: the characteristic variables of the jth region (the regional provinces, urban and rural classification variables, as the second layer variables).

Secondly, the first layer model (individual level) is established. The first layer model describes the direct impact of socioeconomic status on the allocation of educational resources. The specific mathematical Equation is established as follows:


$$Y_{ij}=\beta_{0j}+\beta_{1j}X_{ij}+r_{ij}$$ (7)

In Equation (7), $\beta_{0j}$ is the intercept of the jth region (or urban and rural), indicating the expected value of educational resource allocation in the region when the socioeconomic status is 0. $\beta_{1j}$ is the slope of the jth region (or urban and rural), indicating the impact of socioeconomic status on the allocation of educational resources. $r_{ij}$ is the residual of the first level, which represents the partial variation that is not explained by the first level model.

Then, a second-level model (region and urban-rural level) is established. The second-level model introduces region and urban-rural classification as high-level variables to explain the variation of intercept and slope in the first-level model. The specific mathematical Equation is established as follows:


$$\beta_{0j}=\gamma_{00}+\gamma_{01}Z_{j}+u_{0j}$$ (8)

$$\beta_{1j}=\gamma_{10}+\gamma_{11}Z_{j}+u_{1j}$$ (9)

In Equation (8) and Equation (9), $\gamma_{00}$ and $\gamma_{10}$ are the second-level intercepts, which represent the expected values of the intercept and slope of the first-level model when the regional characteristic variable is 0, respectively. $\gamma_{01}$ and $\gamma_{11}$ are the slopes of the second layer, indicating the degree of influence of regional characteristic variables on the intercept and slope of the first layer model. $u_{0j}$ and $u_{1j}$ are residuals at the second level, representing partial variations that are not explained by the second level model.

Finally, the two-layer model is combined to obtain the following:


$$Y_{ij}=(\gamma_{00}+\gamma_{01}Z_{j}+u_{0j})+(\gamma_{10}+\gamma_{11}Z_{j}+u_{1j})X_{ij}+r_{ij}$$ (10)

Through this method, we can more accurately understand the complex relationship between family background, socioeconomic status, regional and urban-rural differences, and the allocation of educational resources, and can consider the influencing factors at the individual level and the group level at the same time, so as to provide a more comprehensive analysis perspective. Comparing the effects of different levels can more accurately estimate and explain the specific impact of regional and urban-rural differences on the allocation of educational resources.

## 3 Results

### 3.1 Descriptive results

**Fig 2:**
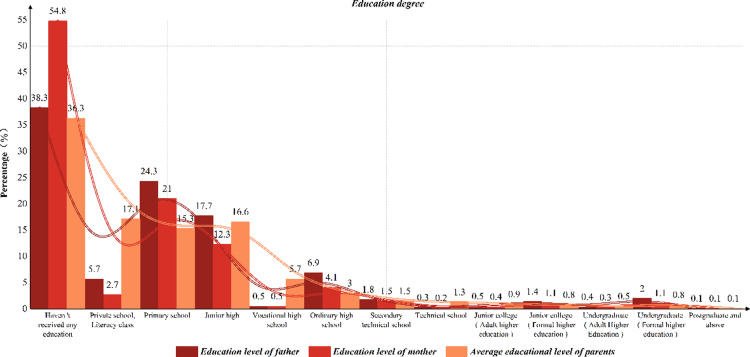
Statistics of parents’ education level.

First of all, we make a detailed statistics on the education level of parents in the sample. The results show that most parents’ education level is concentrated in the middle and low education level, and the proportion of parents with higher education is relatively low. This distribution reflects the overall situation of the current family education background in China.Although there is a certain educational foundation, high-level education is still relatively scarce. The specific statistical data are shown in Fig 2.

**Fig 3:**
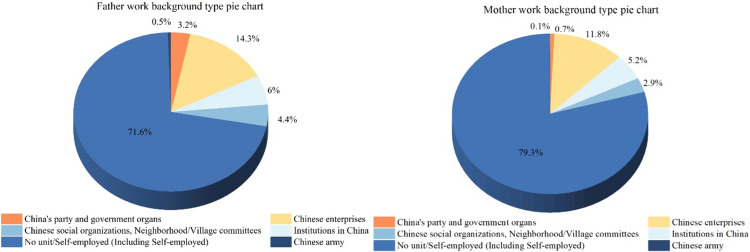
Pie chart of parents’ work background statistics.

Secondly, we classify and count the work background of parents. The results show that parents have diverse occupational backgrounds, but in the case of a large population in China, parents without unit/ self-employment (including self-employed) account for the largest proportion. This diversity reflects the complexity of China ‘s social and economic structure. The details are shown in Fig 3.

**Fig 4:**
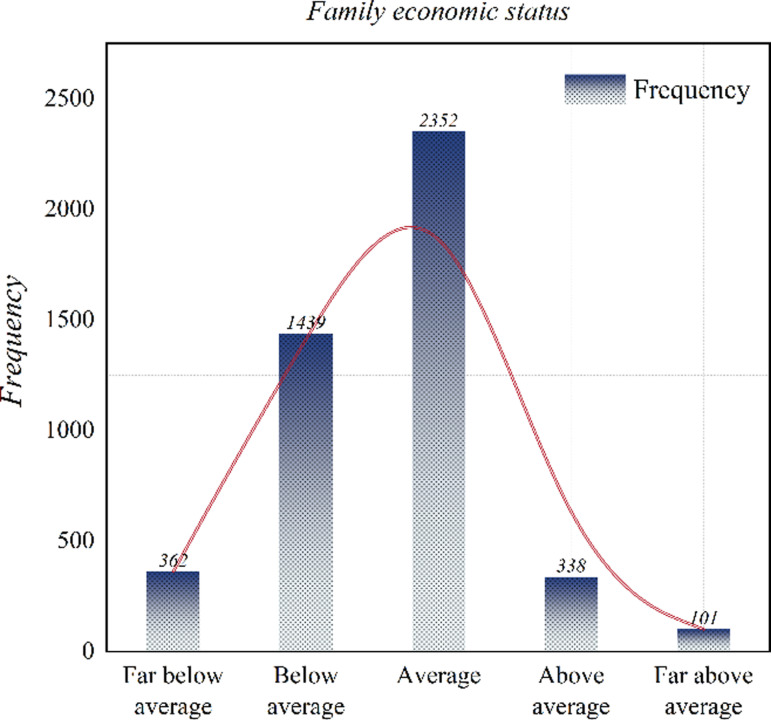
Family economic status statistics.

Again, we also made statistics on the family’s economic situation. We find that the overall economic situation of the sample families shows a normal distribution, that is, most families are at a medium economic level, and a very small number of families are at a very high or very low economic level. The results show that the differences in the economic status of different families may have different degrees of impact on the education level of children. The specific statistical data are shown in Fig 4.

**Fig 5:**
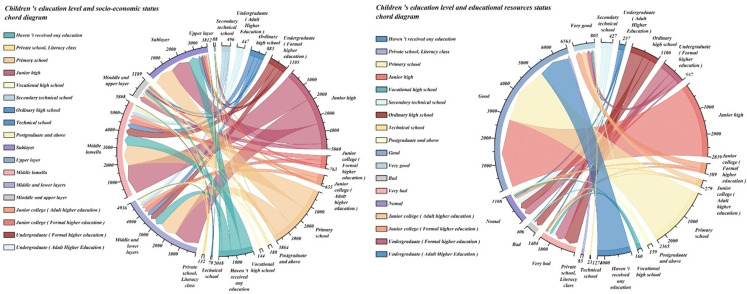
children’s education level and socioeconomic status, educational resources chord diagram.

Finally, we conducted a statistical analysis of children’s education level, socioeconomic status and educational resources. Using the string diagram, we can see that children from families with higher socioeconomic status tend to achieve higher educational levels, although regional and other contextual factors may influence this relationship. In terms of children’s education level, we can also see that there is a close relationship between education resources and children’s education level. In the case of good education resources, children’s education level has generally increased by 20% ~ 30%. However, we also find that in the case of poor educational resources, children also receive higher education, which also shows that in China ‘s current educational environment, the education of children in Chinese families can receive higher education, reflecting that China’s educational resources are not completely unfair. The specific statistical results are shown in Fig 5.

In summary, we have a preliminary understanding of the basic characteristics of the sample families in terms of parental education, work background, and family economic status. In the following analysis, we will further explore the influence mechanism of family background on children’s education level, and whether socioeconomic status has a moderating effect.

### 3.2 Results of principal component analysis model and hierarchical regression model

As shown in Table 1, the value of KMO is 0.865. At the same time, the results of Bartlett’s spherical test showed that the significant P value was 0.000, which was significant at the level, indicating that there was a correlation between the variables. KMO and Bartlett ‘s tests show that the results of principal component analysis are valid.

**Table 1:** Test results of KMO and Bartlett.

KMO test and Bartlett test		
KMO value		0.865
Bartlett sphericity test	Approximate chi-square	1533.899
	Degree of freedom	3
	Predominance	0.000

**Fig 6:**
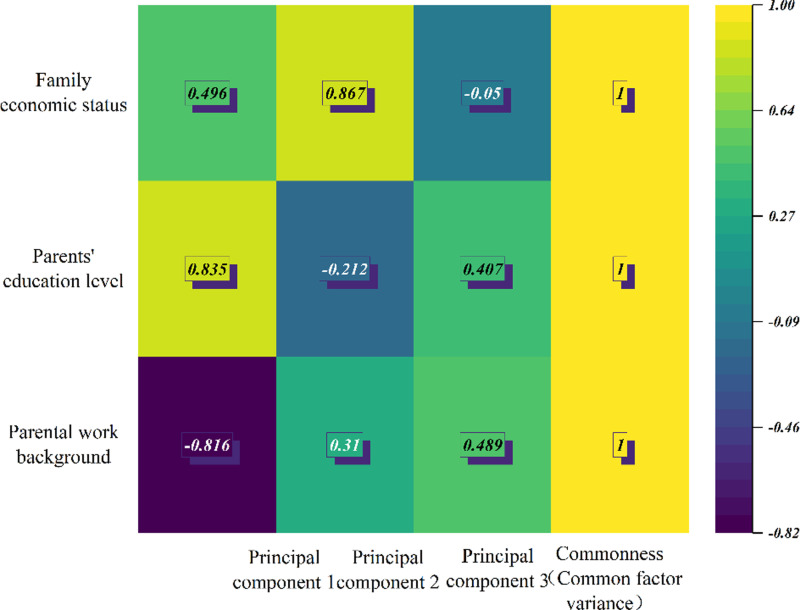
Factor load matrix heat map.

**Fig 7:**
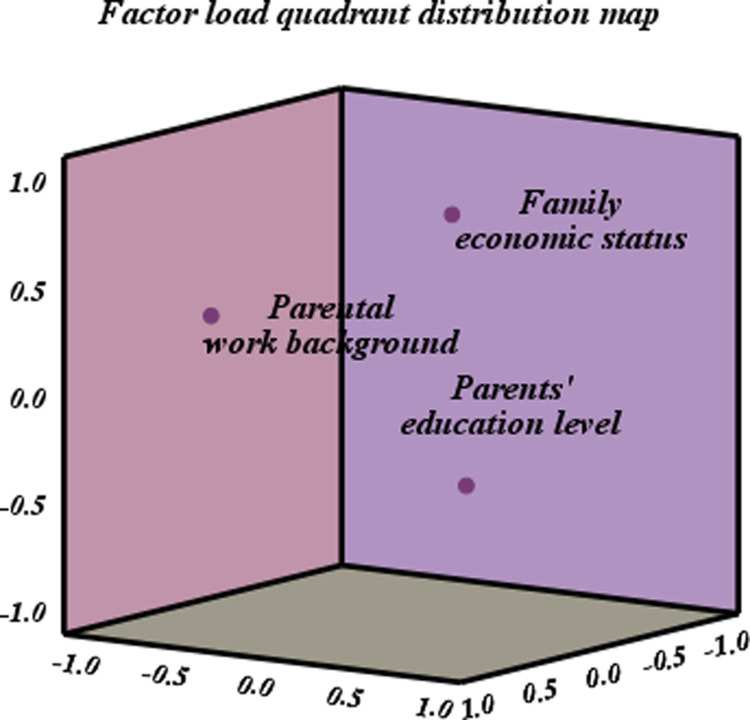
Factor load quadrant distribution diagram.

Because this study uses the information of the three original variables of parents’ education level, parents’ working background and family economic status to reflect the comprehensive variable of family background, this study extracts all three principal components. The results of factor loading coefficient are shown in Table 2, the thermal condition of factor loading matrix and the quadrant distribution of factor loading are shown in Fig 6 and Fig 7.

**Table 2:** Factor load coefficient results table.

Variable	Factor load coefficient			Commonness
	Principal component 1	Principal component 2	Principal component 3	
Family economic status	0.496	0.867	-0.05	1
Parents’ education level	0.835	-0.212	0.507	1
Parental work background	-0.816	0.31	0.489	1

Then, according to the factor score coefficient, the component score is calculated, and the factor formula is obtained. The component score results and the principal component weight results are shown in Table 3 and Table 4.

**Table 3:** Component matrix table.

Variable	Component 1	Component 2	Component 3
Family economic status	0.308	0.971	-0.101
Parents’ education level	0.519	-0.238	1.017
Parental work background	-0.507	0.347	0.98

**Table 4:** Principal component weight results table.

Variable	Variance explained rate	Cumulative variance interpretation rate	Weight
Component 1	0.536	53.634%	53.634%
Component 2	0.297	83.382%	29.749%
Component 3	0.166	100%	16.618%

As shown in the results of Table 3 and Table 4, the comprehensive score of the new variable of family background is calculated. The calculation Equation is as follows:


$$F=\left(\frac{0.536}{1}\right)\times F1+\left(\frac{0.297}{1}\right)\times F2+(\frac{0.166}{1})\times F3$$ (11)

$$\left\{ \begin{array}{c} F1=0.308*X3+0.519*X1-0.507*X2\\ F2=0.971*X3-0.238*X1+0.347*X2\\ F3=-0.101*X3+1.017*X1+0.98*X2 \end{array}\right.\;$$ (12)

In order to explore whether family background affects children’s education level? And does socioeconomic status have a moderating effect in family education? This study constructs a basic regression model to analyze the direct impact of family background on children’s education level.

**Fig 8:**
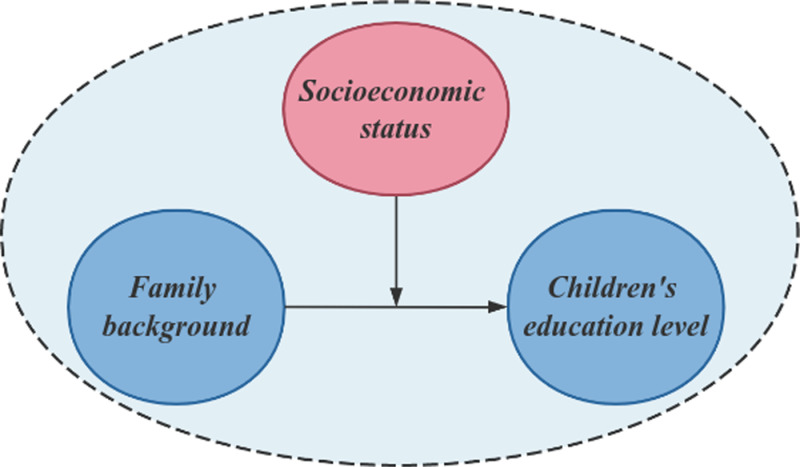
Regulatory effect mechanism diagram.

**Fig 9:**
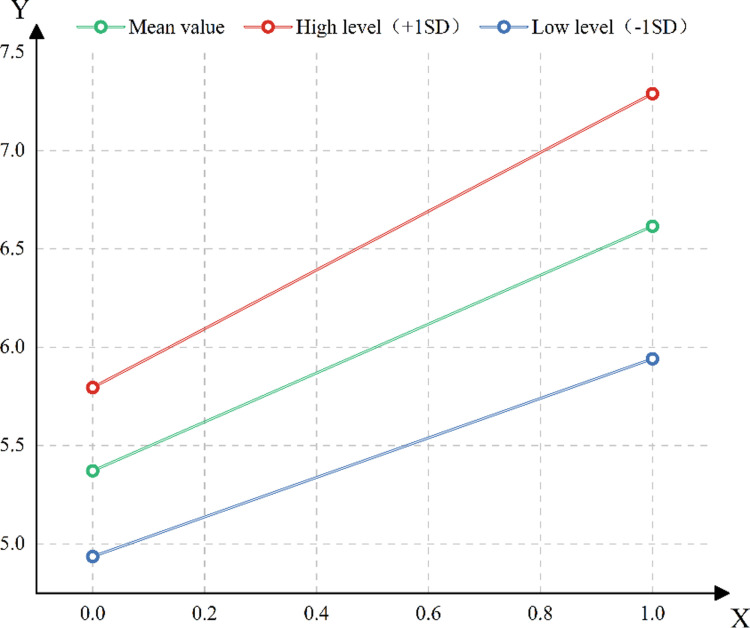
Difference slope diagram.

As shown in Fig 8, the dependent variable Y: {children’s education level}; independent variable X: {family background}; moderating variable M: {socioeconomic status}. After calculation, the results of the adjustment effect are shown in Table 5, Table 6 and Table 7, and the difference of the amplitude of X to Y at different levels (low, medium and high) of the adjustment variable M is shown in Fig 9.

**Table 5:** Moderating effect results (Model 1).

Dependent variable：children’s education level				
	Coefficient	Standard error	t	P
Const	5.385	0.049	109.867	0.000
Family background	1.266	0.077	16.407	0.000
R^2^	0.655			
Adjust R^2^	0.655			
F	F(4592，1)=269.205，P = 0.000			
△R²	0.655			
△F	△F(1，4592)=269.205，P = 0.000			

**Table 6:** Moderating effect results (Model 2).

Dependent variable：children’s education level				
	Coefficient	Standard error	t	P
Const	4.373	0.131	33.447	0.000
Family background	1.225	0.077	15.963	0.000
Socioeconomic status	0.437	0.052	8.34	0.000
R^2^	0.670			
Adjust R^2^	0.669			
F	F(2，4589)=171.387，P = 0.000			
△R²	0.670			
△F	△F(1，4589)=69.548，P = 0.000			

**Table 7:** Moderating effect results (Model 3).

Dependent variable：children’s education level				
	Coefficient	Standard error	t	P
Const	4.35	0.131	33.263	0.000
Family background	0.602	0.206	2.923	0.003
Socioeconomic status	0.443	0.052	8.444	0.000
Family background* Socioeconomic status	0.274	0.084	3.252	0.001
R^2^	0.672			
Adjust R^2^	0.671			
F	F(3，4588)=118.021，P = 0.000			
△R²	0.672			
△F	△F(1，4588)=182.319，P = 0.000			

From Table 5, Table 6 and Table 7, it can be seen that the hierarchical regression model verifies the direct impact of family background on children’s education level and its interaction with the moderating variable socioeconomic status (SES). Model 1 shows that family background has a significant positive impact on children’s education level (*β*=1.266; *p* < 0.001); after introducing the variable of socioeconomic status in model 2, the independent effect of socioeconomic status is also significant (*β*=0.437; *p* < 0.001); model 3 further adds the interaction term (family background × socioeconomic status), and its coefficient is significantly positive (*β*=0.274; *p* < 0.001), indicating that socioeconomic status has a positive moderating effect on the relationship between family background and education level.

As shown in Fig 9, it intuitively shows the dynamic mechanism of the moderating effect: under the high socioeconomic status level, the slope of the impact of family background on education level is steeper, indicating that high socioeconomic status families can significantly amplify the advantages of the original family background through economic resources (such as high-quality extracurricular tutoring, international education opportunities) and social capital (such as occupational networks, policy resource tilt). For example, even if parents’ education level is low, families with high socioeconomic status can still make up for their disadvantages through economic investment, or obtain scarce educational resources through social relations. On the contrary, the slope of the low socioeconomic status group is relatively flat, and the positive effect of family background is suppressed. Even if parents are highly educated, they are limited by economic constraints or lack of social capital, and it is difficult to convert family cultural advantages into actual education investment (such as inability to bear extracurricular training costs or unable to obtain admission qualifications for elite schools), resulting in limited improvement of children’s education level. We can conclude that when the family background affects the children’s education level, the adjustment variable socioeconomic status has a significant difference in the range of influence at different levels, and when the family background affects the children’s education level, the socioeconomic status plays a positive regulatory role.

### 3.3 Hierarchical linear model results

**Fig 10:**
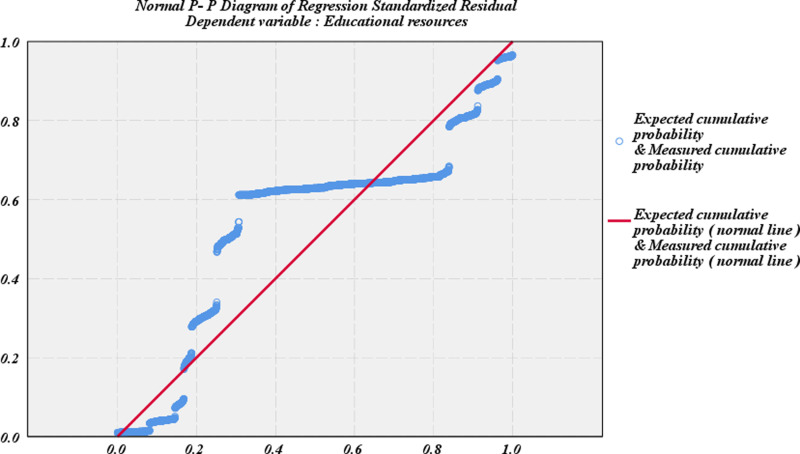
Regression standardized residual normal P-P plot.

Firstly, the allocation of educational resources is taken as the dependent variable. In the first-level model, socioeconomic status is introduced as an independent variable. Secondly, in the second layer model, the provincial regions and urban and rural areas are introduced as high-level variables to capture the group effects of these variables on the allocation of educational resources. Finally, according to the regression results, the effects of each level, especially the second level, are analyzed to judge whether the regional and urban-rural differences affect the distribution of educational resources. The results of the hierarchical linear model are shown in Table 8, and the normal probability of regression standardized residuals is shown in Fig 10.

**Table 8:** Hierarchical linear model results table.

	Variable	Satisfaction	
		Model 1	Model 2
First floor	Socioeconomic status	0.491	0.290
Second floor	Region	–	-0.022
	Urban and Rural	–	3.041
	F	83.359	412.440
	R²	0.718	0.768
	△R²	0.718	0.750

As shown in Table 8 and Fig 10, both Model 1 and Model 2 were statistically significant (F1 = 83.359, F2 = 412.44, both P1 and P2 < 0.001). According to the change of R², R² is 0.718 in Model 1.After adding regional and urban-rural variables, R² becomes 0.768 in Model 2.The explanatory power of the model has been greatly improved, indicating that regional and urban-rural differences have a significant impact on the distribution of educational resources in China. Therefore, we can prove that under different socioeconomic status levels, the differences between urban and rural areas in China significantly affect the distribution of educational resources in China.

## 4 Discussion

### 4.1 Family background has a significant positive impact on children’s education level

Our results confirm that family background has a significant positive effect on children’s education level, supporting previous studies [34,35]. This multidimensional view aligns with prior research on socioeconomic factors. In detail, the level of education of parents often determines their emphasis on children’s education, the choice of education methods and the richness of educational resources that can be provided for their children. parents’ working background is directly related to the family’s economic status and social status, which in turn affects the investment and opportunities in children’s education [36]. As a basic factor, family economic status directly restricts the family’s ability to spend on education, such as tuition fees, tutoring fees, and extracurricular activities. After clarifying the influence of family background factors on children’s education level, we find that these effects are not isolated, but intertwined and work together [37]. For example, families with highly educated parents tend to pay more attention to their children’s education, which can provide better learning environment and resources for their children; at the same time, parents in these families are also more likely to gain more educational opportunities for their children through their own social communication networks [38]. The existence of this multiple influence makes the influence of family background on children’s education level complex and multidimensional. These family background elements do not play a role in isolation, but are intertwined and synergistic. Families with highly educated parents usually create a strong learning atmosphere. This family culture will subtly affect their children and make them have a positive attitude towards learning from an early age. At the same time, parents in these families rely on their social communication networks to win more high-quality educational resources and opportunities for their children, which further strengthens the positive impact of family background on their children’s education level. For example, parents are the families of college teachers. Children grow up in the campus environment from a young age and are influenced by the academic atmosphere. Parents can also use work convenience to provide children with opportunities to participate in scientific research projects. The superposition of multiple effects makes the impact of family background on children’s education level present complexity and multidimensionality.

In this study, although the family background is studied, the family background is a multi-dimensional concept. In the follow-up study, more measurement parameters for the variable of family background, such as family cultural background, should be included. At the same time, for future research, it is hoped that the consideration of time dimension will be added to the research variables and contents, which will be more conducive to the rigor and foresight of this research.

### 4.2 Socioeconomic status has a significant positive moderating effect on family background and children ‘s education level

The results show that socioeconomic status has a significant positive moderating effect on the relationship between family background and children’s education level (model 3 interaction coefficient *β*=0.274; *p* < 0.001), which verified hypothesis 2. The moderating effect is presented by the significance of the interaction term and the difference slope plot (as shown in Fig 8 above). When the family’s socioeconomic status (SES) is at a high level, the positive impact of family background on children’s education level is significantly enhanced; when the socioeconomic status is at a low level, the positive effect of family background is suppressed. For example, in high socioeconomic status families, for every 1 unit increase in parental education, the level of children’s education can increase by 1.5 units (slope of high socioeconomic status group), while only 0.7 units in low socioeconomic status group, and the intensity of adjustment effect is significantly different (*t* = 3.252, *p* < 0.001). As an impor*t*ant aspect of family background, socioeconomic status itself contains rich social resources and opportunities. In high socioeconomic status families, children not only enjoy better educational resources, but also have access to wider social networks and more development opportunities [39]. These advantages make them in a more advantageous position in the education competition, which makes it easier to obtain a high level of educational opportunities [40]. On the contrary, in families with low socioeconomic status, children face more difficulties and challenges in education due to the lack of resources and opportunities [41]. This inequality of social and economic status may further aggravate the inequality of education in China.

It is worth noting that the internal mechanism of the moderating effect is reflected in the role of socioeconomic status through the two paths of resource leverage effect and structural opportunity transformation. First of all, families with high socioeconomic status can transform the potential advantages of family background into actual educational resources. For example, if families with highly educated parents have high socioeconomic status at the same time, they can make up for the lack of family cultural capital through economic capital (such as purchasing international courses, hiring professional teachers) and social capital (such as using professional networks to obtain high-quality school enrollment), thus significantly improving their children’s education level. On the contrary, families with low socioeconomic status, even if their parents have higher education, may be diluted by the positive effects of family background due to economic constraints (such as inability to pay for after-school tutoring) or social network limitations (such as lack of recommendation channels for further education). This differentiation is further verified in the Hierarchical Linear Model (HLM): the marginal effect of family background in high socioeconomic status families in eastern urban areas is 32% higher than that in rural areas in the central and western regions, indicating that the concentration of regional educational resources strengthens the moderating effect of socioeconomic status.

At the same time, in this study, socioeconomic status does not pass on the influence of family background through mediating variables such as family education methods or parental expectations (the independent effect of socioeconomic status in Model 2 *β*=0.437 does not weaken the coefficient of family background *β*=1.225), but directly changes the boundary conditions of the relationship between family background and education level. This finding suggests that policy intervention should focus on weakening the moderating strength of socioeconomic status, such as narrowing the difference in access to educational resources between families with high and low socioeconomic status through fiscal transfer payments, rather than only targeting the independent role of family background or socioeconomic status. For example, the design of targeted education subsidies for low socioeconomic status families can help them break through economic barriers and activate the potential advantages of family backgrounds; by guiding the educational resources investment of high socioeconomic status families through policies (such as requiring high-quality schools to reserve places for poor students), the resource monopoly caused by the adjustment effect can be suppressed. This conclusion has important implications for education equity policy: it is difficult to eliminate education inequality by simply improving the independent effect of family background or socioeconomic status, and the differentiation effect of the interaction between the two must be weakened through structural reforms.

Finally, the moderating effect of socioeconomic status is not limited to the direct impact of family background on children’s education level. For example, in families with high socioeconomic status, parents tend to have higher educational expectations for their children and are able to provide their children with richer educational resources and more learning opportunities; in families with low socioeconomic status, due to the limitation of parents’ own education level and economic ability, they may have relatively low expectations for their children’s education and relatively few educational resources and opportunities for their children. Moreover, parents’ own life experience and cognitive level will also affect their ability to guide their children’s education, which makes children lack effective guidance in learning methods and development direction, and further widens the gap between children with different family backgrounds in education level. This difference in family education environment and expectations caused by different socioeconomic status may further aggravate the gap in the level of education of children with different family backgrounds. In such an environment, children face more difficulties and challenges in the process of education, such as learning difficulties are difficult to be solved in a timely and effective manner, and they are confused about their future career planning and development direction, which further aggravates the phenomenon of educational inequality in China.

### 4.3 Regional and urban-rural differences affect the allocation of educational resources

The results show that regional and urban-rural differences affect the allocation of educational resources, which verifies hypothesis 3. In China, a country with a vast territory and a large population, the imbalance between regional and urban-rural development is a long-standing problem. This imbalance is not only reflected in the level of economic development, but also in the distribution of educational resources.

In terms of regional differences, there are obvious differences in the level of economic and social development, investment in educational resources and policy support among different regions. Generally speaking, developed regions or large cities tend to have richer educational resources and higher quality of education, which can provide better educational opportunities and conditions for children. On the contrary, in less developed areas or small towns, rural areas, due to the lack of educational resources and insufficient investment, children are unlikely to face more educational opportunities and educational resources [42–44]. This regional difference has an important impact on children’s education level and future development. In terms of urban and rural differences, there are also significant differences in the allocation of educational resources and the quality of education between urban and rural areas. Generally speaking, urban areas have richer educational resources and higher quality of education, which can provide better educational opportunities and conditions for children. On the contrary, in rural areas, children may face more educational opportunities and resource constraints due to problems such as lack of educational resources and insufficient investment [45,46]. This uneven distribution of educational resources between urban and rural areas not only aggravates the phenomenon of educational inequality, but also limits the development potential of children in rural areas and poor areas. At the same time, in order to better understand the impact of regional and urban-rural differences on the allocation of educational resources, this study further discusses the macro and micro levels.

At the macro level, national and local government education policies, financial investment and education resource allocation mechanisms have an important impact on the allocation of educational resources between regions and urban and rural areas [47,48]. For example, some regions have received more investment in educational resources due to historical reasons or policy preferences, thus forming a relatively superior educational environment; other regions may be in a state of lack of educational resources for a long time due to factors such as remote geographical location and weak economic foundation. At the micro level, the differences in teachers, teaching facilities and curriculum between schools also directly affect the level of education of students [49,50]. In some high-quality schools, students have access to more advanced teaching concepts and methods, more extracurricular activities and more development opportunities [51]; in some schools with poor conditions, students may face problems such as insufficient teachers and backward teaching facilities.

## 5 Conclusions

Through principal component analysis model, hierarchical regression model and hierarchical linear model, this study explores the influence of family background and socioeconomic status on children’s education level, and draws the following conclusions: (1) Family background and socioeconomic status can affect children’s education level; (2) Family background can directly affect children’s education level, and through the adjustment of socioeconomic status; (3) Regional and urban-rural differences significantly affect the allocation of educational resources. Based on this, we suggest that: First, Targeted investments should focus on improving access to quality education in under-resourced regions. This includes increasing funding for teacher training, enhancing infrastructure, and implementing financial aid programs for low-income families. Second, efforts should be made to break down the barriers to the allocation of educational resources between regions and urban and rural areas, and promote the balanced distribution of educational resources between different regions and urban and rural areas. This includes measures such as strengthening the construction of educational infrastructure, optimizing the allocation of teacher resources, and improving the education financial transfer payment system. Third, we should pay attention to the important role of family background in children’s education, and encourage families to create a good educational environment for their children through policy guidance and support.
